# Surface Warming During the 2018/Mars Year 34 Global Dust Storm

**DOI:** 10.1029/2019GL083936

**Published:** 2020-05-06

**Authors:** Paul M. Streeter, Stephen R. Lewis, Manish R. Patel, James A. Holmes, David M. Kass

**Affiliations:** ^1^ School of Physical Sciences The Open University Milton Keynes UK; ^2^ Space Science and Technology Department Science and Technology Facilities Council, Rutherford Appleton Laboratory Didcot UK; ^3^ Jet Propulsion Laboratory California Institute of Technology Pasadena CA USA

**Keywords:** Mars, Mars atmosphere, dust storm, data assimilation, Mars Climate Sounder

## Abstract

The impact of Mars's 2018 Global Dust Storm (GDS) on surface and near‐surface air temperatures was investigated using an assimilation of Mars Climate Sounder observations. Rather than simply resulting in cooling everywhere from solar absorption (average surface radiative flux fell 26 W/m^2^), the globally averaged result was a 0.9‐K surface warming. These diurnally averaged surface temperature changes had a novel, highly nonuniform spatial structure, with up to 16‐K cooling/19‐K warming. Net warming occurred in low thermal inertia regions, where rapid nighttime radiative cooling was compensated by increased longwave emission and scattering. This caused strong nightside warming, outweighing dayside cooling. The reduced surface‐air temperature gradient closely coupled surface and air temperatures, even causing local dayside air warming. Results show good agreement with Mars Climate Sounder surface temperature retrievals. Comparisons with the 2001 GDS and free‐running simulations show that GDS spatial structure is crucial in determining global surface temperature effects.

## Key Points

- Mars's 2018 Global Dust Storm caused a 0.9‐K globally averaged surface warming but with local 16‐K cooling/19‐K warming
- The magnitude of dayside cooling was controlled by atmospheric dust, and nightside warming by surface thermal inertia
- The effects were strongly nonuniform, with high dust loading causing net warming (cooling) over low (high) thermal inertia continents

## 1 Introduction

Dust aerosol is a critical component of Mars's atmosphere and has long been known to have significant radiative and dynamical effects through scattering and absorption of radiation (e.g., Gierasch & Goody, 1972; Pollack et al., 1979). Global dust storms (GDS; here, events spanning all longitudes over a wide range of latitudes) are a spectacular example of dust‐related phenomena on Mars, occurring every few Martian years (MYs) and covering swathes of the planet with a deep dust cloud for months at a time (e.g., Haberle, 1986; Leovy et al., 1973; Zurek, 1982; Zurek & Martin, 1993). These storms have been modeled to have substantial effects on the circulation (e.g., Böttger et al., 2004; Bougher et al., 1997; Haberle et al., 1982; Lewis & Read, 2003) and radiative balance (e.g., Read et al., 2016) of the atmosphere.

One way to describe the degree of dust loading in the atmosphere is by optical depth, defined as the log of the ratio of incident to transmitted intensity of a beam at a certain wavelength (Petty, 2006). In practice, the radiative effects of an atmospheric aerosol also depend on particle radius and its specific scattering/absorption properties. Dust generally has a greater scattering effect on incident sunlight than smoke (which is compositionally different and, on Earth, generally smaller; Friedlander, 2000), which primarily absorbs in the visible; smoke therefore has a greater “anti‐greenhouse effect,” and it has been famously theorized that a global smoke cloud on Earth would result in drastic surface cooling (“nuclear winter”; Turco et al., 1984), with soot/smoke used in nuclear winter simulations of single‐scattering albedo (SSA; ratio of scattering to extinction at solar wavelengths) of, for example, 0.64 (Robock et al., 2007). Soil dust alone has been shown to have surface radiative effects that are highly dependent on the specific SSA used, with higher SSAs (0.97 vs. 0.84) causing less shortwave flux reduction at the surface (Shell & Somerville, 2007). Recent work on the properties of Martian atmospheric dust, based on observations of the 2007 GDS, estimates a SSA of 0.94 (Wolff et al., 2009), significantly greater than that of soot/smoke. Aerosol properties are critical for determining aerosol radiative effects, and SSA in particular has a large impact on shortwave radiative flux at the surface.

High opacities in a Mars global circulation model have been shown to decrease surface shortwave flux while increasing longwave emission to the surface, with a net reduction in surface flux of ~70 W/m^2^ as averaged over an MY for the unrealistic scenario of a visible‐wavelength opacity 5 dust cloud covering the planet for a whole orbital cycle (Read et al., 2016). In situ observations of the 2018 GDS from the Mars Science Laboratory (MSL) showed substantial dayside surface and near‐surface cooling due to the reduction in shortwave flux but also a nightside warming effect (Guzewich et al., 2019), this latter effect due to enhanced longwave emission and backscattering as a result of the increased aerosol and consequently higher atmospheric temperatures (Martínez et al., 2017). Orbital measurements confirm this (see section 4).

Surface properties have also been shown to be key in determining surface temperatures (STs) and near‐surface air temperatures (ATs). The surface thermal inertia (TI) describes the temperature response of the surface to incident energy flux and is especially important on Mars given the low atmospheric density and lack of oceans to act as heat reservoirs. Materials with low TI, such as loosely aggregated dust, heat and cool rapidly, while materials with high TI (like bedrock) stay relatively warm at night and cool in the day. Ground temperatures at the MSL site, for example, are driven mostly by the local TI, with lower (higher) TI regions resulting in more (less) extreme minimum and maximum ground temperatures (Martínez et al., 2017). These lower nightside/higher dayside temperatures at low‐TI regions are due to increased radiative heating on the dayside and rapid radiative cooling on the nightside.

## 2 Methods

### 2.1 Model

The Mars Global Circulation Model (MGCM) is a four‐dimensional numerical model that is the result of a collaborative effort between the Laboratoire de Météorologie Dyamique, the University of Oxford, the Open University, and the Instituto de Astrofísica de Andalucía (Forget et al., 1999). The version used here contains a spectral dynamical core with a finite‐difference scheme in the vertical and a semi‐Lagrangian advection scheme (Lewis et al., 2007). Dust is advected by the MGCM using a two‐moment scheme with a log‐normal size distribution (of representative mean effective radius ~1 μm; Madeleine et al., 2011) with total column dust optical depths (CDOD) scaled to match assimilated observations (supporting information Text S1). The vertical dust distribution was allowed to evolve freely. Model dust is radiatively active, with radiative properties derived from observational work (Wolff et al., 2006, 2009; see Figures S4 and S5 for uncertainties in SSA). Unless specified, all opacities described in the context of the MGCM are true CDOD at 600 nm. The MGCM was run using a spectral resolution of T42, corresponding to a spatial resolution of ~3.75° (~215 km at the equator), and with 50 vertical levels at constant pressure/surface pressure, with midpoints ranging from ~5 m to ~105 km above the surface. The water cycle parametrizations were not included in order to isolate the effects of dust; besides, the greatest radiative effects of water occur in the aphelion season (Steele et al., 2014). The MGCM includes a detailed TI map derived from orbital measurements (Putzig et al., 2005).

### 2.2 Mars Climate Sounder Data and Assimilation Technique

The data assimilation scheme used is a version of the Analysis Correction scheme, created by the UK Met Office for operational use on Earth (Lorenc et al., 1991) and modified for the Martian atmosphere (Lewis et al., 1997; Lewis et al., 2007). Temperature profiles are assimilated in the same manner as previously used for Thermal Emission Spectrometer (TES; Holmes et al., 2018; Lewis et al., 2007) and Mars Climate Sounder (MCS; Holmes, Lewis, Patel, & Smith, 2019; Steele et al., 2014) data, while dust is assimilated spatially in the form of columns (Lewis et al., 2007). See Text S2 for further details.

The two assimilated fields, temperature profiles and dust column products, are from MCS, a limb sounding instrument aboard the Mars Reconnaissance Orbiter. Temperature and dust retrievals extend to altitudes of ~85 km, with an intrinsic vertical resolution of ~5 km (McCleese et al., 2010), though the MY 34 GDS led profiles to start and end at higher‐than‐usual altitudes for the GDS period. Mars Reconnaissance Orbiter's Sun‐synchronous orbit means observations are made at two local times, 0300 and 1500 in nonpolar regions (Kleinböhl et al., 2009). Quality control applied to dust retrievals is described in Text S3 (see also Montabone et al., 2020). Before assimilation into the MGCM, dust opacities are converted from 21.6 μm to 600 nm via a conversion factor of 7.3 (Kleinböhl et al., 2011). The retrievals used are the most recently processed available (Kleinböhl et al., 2017). The retrievals used for the GDS itself are v5.3.2.

### 2.3 Simulations Performed

Two MGCM simulations with data assimilation (“reanalyses”) were performed, for MY 30 and 34, in addition to 15 free‐running MGCM simulations; all relevant data are freely available on the Open Research Data Online (ORDO) repository (link: Streeter et al., 2019). The reanalyses assimilated MCS 3‐D temperatures and 2‐D CDOD. MY 30 was chosen because of its relative lack of major dust activity. A preexisting reanalysis of the MY 25 GDS was also used (Holmes, Lewis, & Patel, 2019), using TES temperatures and column dust (e.g., Montabone et al., 2005). The free‐running simulations were made to assimilate artificial dust column data, replicating the start date and rough latitudinal extent (60°S to 40°N) of the 2018 GDS but with prescribed, spatially and temporally uniform CDOD as normalized to the 610 Pa level, ranging from 1 to 15 (the MGCM radiative transfer scheme should be reliable to within ~10% error even at the highest of these; Toon et al., 1989; see Text S4).

## 3 Results

**Figure 1:**
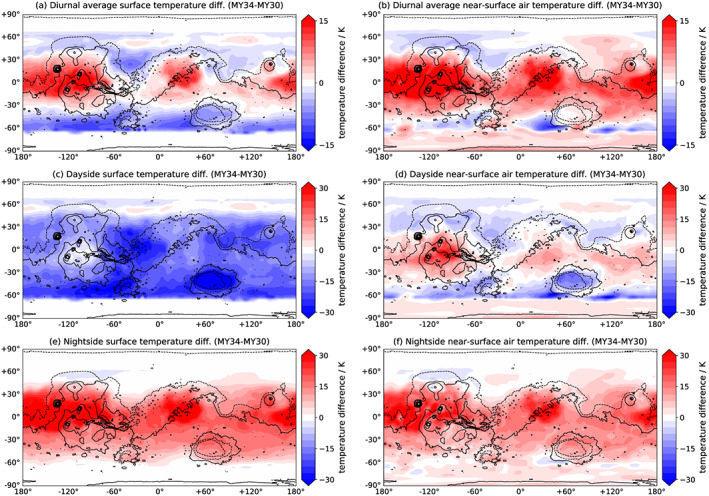
Surface temperature (left) and near‐surface air temperature (right) difference between MY 34 and MY 30 for the period *L*_S_ = 200°–220°: (a, b) diurnally averaged, (c, d) at 1500, and (e, f) at 0300. Solid/dashed contours indicate topography above/below areoid.

Figure 1 displays the diurnally averaged ST difference and the dayside (1500) and nightside (0300) differences during *L*_S_ = 200°–220°, the peak of the 2018 GDS, with local times chosen to match MCS observations. Differences henceforth are in relation to the MY 30 reanalysis; for example, “cooling” is relative to the same period in MY 30. “Global average” refers to the area‐weighted value.

**Figure 2:**
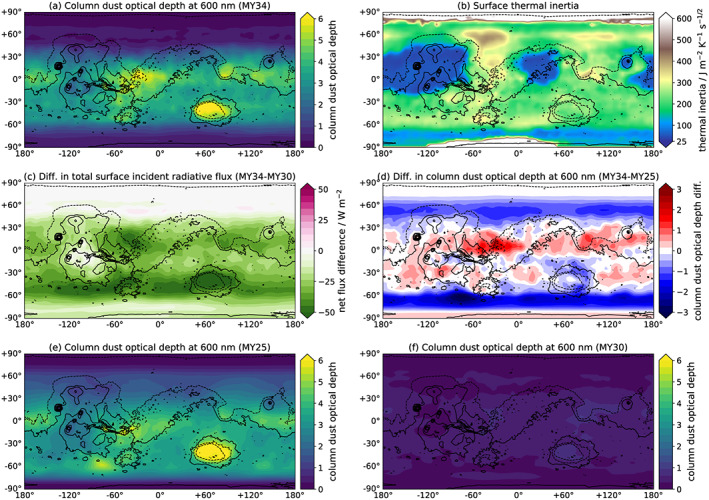
For *L*_S_ = 200°–220°; (a) column dust optical depth in MY 34; (b) surface thermal inertia map used in the Mars Global Circulation Model; (c) diurnally averaged total surface radiative flux difference between MY 34 and MY 30; (d) difference in column dust optical depth between MY 34 and MY 25; (e) column dust optical depth in MY 25; (f) column dust optical depth in MY 30**.**

Mars's dayside surface underwent cooling up to 39 K, global average value 14 K, due to dust‐induced blocking of incident solar radiation. The areas with the greatest cooling include Chryse, northern Hellas, Argyre, Isidis, and Amazonis (Figure 1b). These are all low‐elevation regions and correlate with high dust loading (Figure 2a). Mars's extreme topographic variation means topographic lows have a greater column opacity at the surface than highs, if pressure‐normalized opacities are identical. Low topography regions therefore have higher CDOD. The result was greater cooling over low topography, up to 39 K, and less cooling over high topography, such as the southern highlands and the Tharsis plateau, of <5 K. Note that maximum warming/cooling values are a function of MGCM resolution.

**Figure 3:**
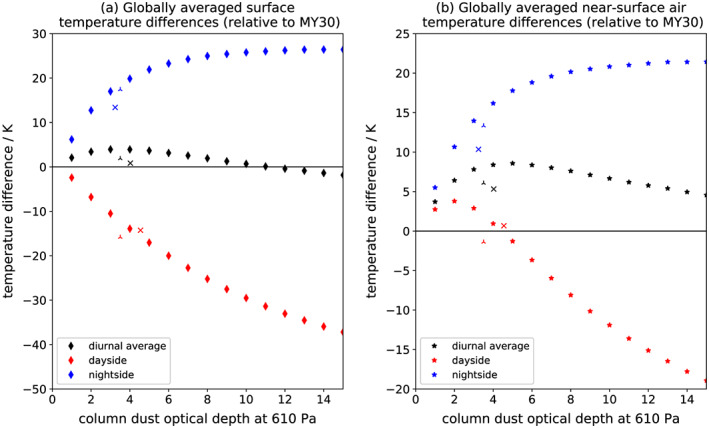
(a) Surface temperature and (b) near‐surface air temperature differences relative to MY 30, globally averaged (area‐weighted) over *L*_S_ = 200°–220° for a range of opacities. Presented are diurnal averages, dayside (1500), and nightside (0300). The MY 34 (MY 25) Global Dust Storm is marked with a cross (three‐pointed star). Column dust optical depths at 610 Pa are also at the relevant local times and are averaged over 60°S to 40°N.

Mars's nightside surface underwent warming of comparable degree to the dayside cooling (Figure 1c), due to the effect of increased backscattering of longwave emission from the surface. This had a magnitude of up to 42 K, with a globally averaged value of 13 K. In contrast to the dayside effects, nightside warming did not correlate with CDOD. This is because the dominant heating effect during the clear‐case Martian night is surface cooling: highly efficient in Mars's thin atmosphere. This cooling rate is driven by surface TI, rather than daytime solar insolation. Therefore, the locations of greatest relative nighttime warming caused by enhanced longwave backscattering are determined by surface TI rather than by CDOD. The warming is greatest at the high‐topography regions of Tharsis and Elysium Mons but also over the low‐elevation Amazonis and Arabia, all low‐TI regions (Figure 3b).

In a globally averaged sense, the nightside 13‐K warming was enough to cancel out the dayside 14‐K cooling; however, as the two were controlled by independent factors—TI and CDOD, respectively—the diurnally averaged effect is not one of exact cancellation. Isidis and the southern highlands show a rough cancellation, but most regions do not (Figure 1a). While there is a net 5‐K cooling over Chryse, the greater effect is a net warming up to 19 K over Amazonis, the low‐TI continents between approximately 160°E and 50°W and between 15°S and 40°N (Amazonis/Tharsis/Elysium), between 0°E and 50°W and between 10°S and 40°N (Arabia Terra), and Elysium Mons (Figure 2b). The global effect of the 2018 GDS was therefore a diurnally averaged increase in STs, due to the strong nightside warming. This was despite a decrease in the diurnal average flux of 10–50 W/m^2^ over most of the planet's surface (Figure 2c).

Globally and diurnally averaged ATs displayed a 5.3‐K increase (Figure 3b). Nightside AT warming closely tracked ST warming in being greatest over low‐TI regions (Figures 1e and 1f); the maximum nightside warming was 37 K. This is because Mars's ATs are mostly surface‐driven. As the nightside surface is warmer during the GDS, so is the nightside near‐surface. Less expected is the dayside near‐surface warming over some regions, where the surface is cooler (Figure 1d). This dayside warming reached up to 31 K over the highest parts of Tharsis. The pattern of dayside warming fell into a latitude band between 10°N and 30°S, corresponding to areas of least dayside surface cooling. This is due to the coupling of STs and ATs caused by dramatically increased absorption of both shortwave and longwave radiation in the atmosphere, a result of the increased dust presence. This, together with the reduced shortwave flux on the surface, significantly reduces the surface‐air temperature gradient. Therefore, despite the dayside ST cooling from the GDS, the decreased surface‐air temperature gradient meant that dayside ATs could be up to 12 K (30 K over Tharsis) higher than in the clear case. If GDS STs were higher than clear ATs, therefore, then so were GDS ATs.

A range of CDOD (normalized to 610 Pa) were tested to explore the impact of greater dust loadings (over the same region/season as the 2018 GDS) on STs and ATs (Figure 3a). Increasing CDOD resulted in increased warming/cooling. However, for CDOD >10 the nightside warming magnitude plateaued, remaining constant at ~25 K due to longwave backscattering reaching its maximum efficiency. By contrast, the dayside cooling magnitude continued to increase with CDOD, albeit at a decreasing rate. This exponential‐like decay follows from the definition of optical depth as the log of the ratio of incident to transmitted flux. The result is global surface warming for CDOD 1–11, peaking at 3.9 K for CDOD 3–4; this range includes the 2018 GDS. For opacities >11 net global cooling resulted, reaching 1.8 K for CDOD 15.

Globally averaged ATs showed a similar pattern to STs, albeit shifted warmer. Nightside ATs exhibited the same plateau as nightside STs, due to the close coupling between the two. Dayside ATs peaked at optical depth 2, which was sufficient to reduce the surface‐air temperature gradient, coupling STs and ATs and thus causing warming, but was sufficiently low that the warming was not outweighed by surface cooling. The diurnally averaged effect was a globally averaged increase in ATs for CDOD 1–15, peaking as an increase of 8.5 K at opacity 5.

**Figure 4:**
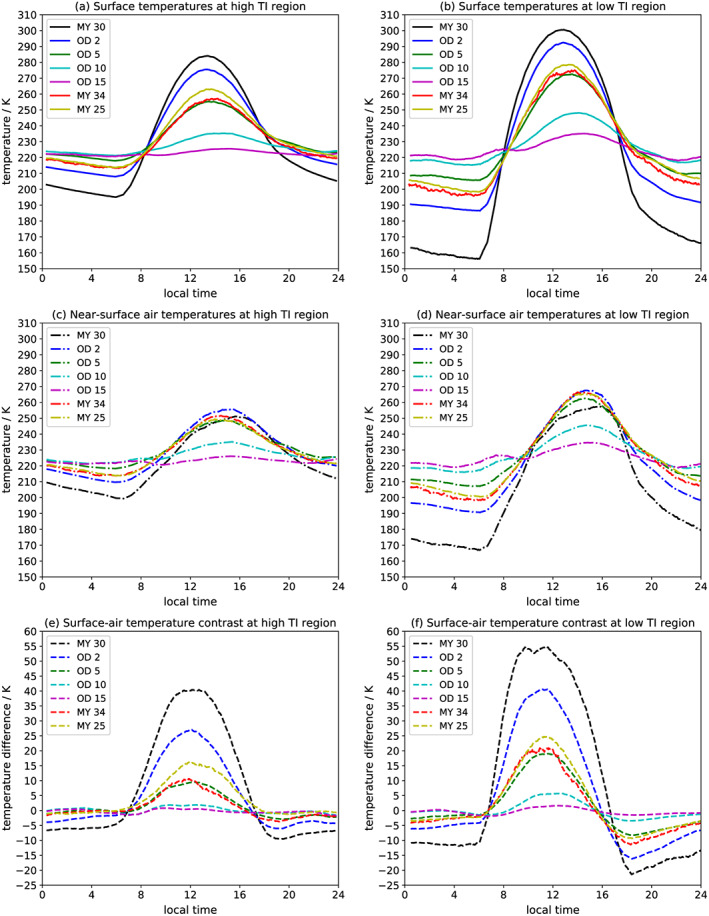
Averaged over *L*_S_ = 200°–220° for (left) a high‐TI region and (right) a low‐TI region: (a, b) surface temperatures, (c, d) near‐surface air temperatures, and (e, f) the surface‐air temperature difference, over the course of a Sol. TI = thermal inertia.

Lastly, we examined ST and AT variation over the course of an average Sol (Figure 4) at a low‐TI (10°N, 30°E) and a high‐TI (5°N, 100°E) location, with near‐identical GDS‐induced radiative flux differences. The differences in the diurnal ST cycle (Figures 4a and 4b) for the clear case are seen in the substantially greater ST variation at the low‐TI region, especially the much colder nightside temperatures from more efficient radiative cooling. The minimum ST rises 18 K (195 to 213 K) in the high‐TI region but 40 K (from 156 to 196 K) in the low‐TI region. Dayside cooling magnitudes are more similar, with maximum ST falling 27 K (284 to 257 K) in the high‐TI region and falling 26 K (301 to 275 K) in the low‐TI region. As discussed, the magnitude of dayside cooling depends on CDOD and reduced shortwave flux rather than surface properties. The overall effect of CDOD >2 is to reduce the diurnal amplitude of both STs and ATs, by nightside warming and dayside cooling, and to reduce the surface‐air temperature gradient, by the coupling mechanism described above. By CDOD 15, the diurnal ST variation decreases from 89 to 5 K (high TI) and from 145 to 17 K (low TI).

A major effect of high CDOD was to dramatically decrease the surface‐air temperature difference on the dayside. For MY 34, the peak surface‐air temperature contrast is 11 and 21 K for the HTI and LTI regions, respectively, compared to 40 and 55 K for MY 30. The nightside surface‐air peak temperature contrast was also reduced from 10 to 4 K (HTI) and from 21 to 11 K (LTI), coupling nightside ATs even more tightly to nightside STs.

## 4 Validation

The MCS surface temperature retrievals of MY 30 and 34 provide an opportunity for validation. As averaged over *L*_S_ = 200–220°, and with MCS' two local times, the retrievals show a globally averaged net ST decrease of 2.1 K, compared to a decrease of 0.9 K from the MY 34 reanalysis using the same local times. Nightside warming agrees very well with the reanalysis on both morphology (greatest warming over low‐TI continents; Figure S1; data are presented with a seasonal CO_2_ cap mask applied. For explanation, see Text S5 and accompanying references: Calvin et al., 2017; Kleinböhl et al., 2020, this issue; McCleese et al., 2008; Piqueux et al., 2015) and in globally averaged value, with warming of 11.2 and 9.1 K for the retrievals and reanalysis, respectively.

Dayside cooling shows greater disagreement, with globally averaged cooling of 15.2 and 11 K, respectively, as well as some disagreement in spatial distribution (Figure S1). The retrievals agree on high cooling over Chryse and Hellas, but also show high (30+ K) cooling over the southern highlands and Amazonis/Elysium Planitia not seen in the reanalysis. There are a number of possible explanations. Error in CDOD is possible, especially at the high values involved (Montabone et al., 2015); however, the results would imply greater CDOD as inferred from the ST retrievals for the dayside but also smaller CDOD on the nightside, that is, a greater diurnal dust variation. Another explanation is SSA differences; the observed difference is greater than that caused by the uncertainty (Figures S5 and S6), but an SSA of 5% difference would be sufficient to cause dayside ST differences of 10+ K (Figure S7). To cause additional cooling specifically, the SSA would have to be in the “dark” part of the observed SSA dichotomy on Mars, contradicting values derived from the 2007 GDS (Wolff et al., 2009). Additionally, the pattern of extra cooling from lower SSA follows locations of greatest CDOD (Figure S7) and thus does not replicate the cooling pattern in the retrievals; invoking SSA would therefore require heterogeneity in the dust population. Another possibility is that the MGCM's particle size scheme (Text S1) underestimates/overestimates particle sizes in particular areas, as with greater lifting occurring during a GDS the particle size structure could be far more heterogeneous than usual (Kahre et al., 2008).

Albedo changes could also play a role: Large‐scale albedo brightening from dust deposition would cause surface cooling by increasing shortwave reflectivity, and if deposition was thin enough this would not necessarily alter TI significantly, explaining the good reanalysis‐retrieval agreement in nightside STs. Finally, there is the question of more systematic and not necessarily GDS‐induced disagreement. While the reanalysis and retrieval nightside STs show very good agreement, there is a systematic dayside bias even in MY 30, a very clear year, of 12 K, going up to 18 K for MY 34 (Figures S2 and S3). Further work is needed to investigate this bias; this may result from MCS limb pointing being affected by topography and affecting surface retrievals, but a full investigation of this is beyond the scope of this work.

Overall, the net ST change shows good morphological agreement with the reanalysis: Average warming is seen over low‐TI continents, average cooling elsewhere. One result of the greater cooling in the retrievals is that the net ST change map displays fewer white regions of little/no ST change; boundaries between areas of net warming/cooling are sharper, showing the important effect of surface TI on the ST response.

TES globally averaged ST retrievals for the 2001 GDS at *L*_S_ = 210° showed a peak dayside cooling of 23 K and a peak nightside warming of 18 K, corresponding to a net decrease of 2.5 K (Smith, 2004). The MY 25 reanalysis shows, for the same time period, a dayside cooling of 21 K and a nightside warming of 16 K, also corresponding to a net decrease of 2.5 K (note that while nightside STs from the MY 25 reanalysis agree well with TES retrievals, there is a systematic ~10‐K disagreement with dayside STs). Averaged over all local times, the reanalysis shows an average ST change of 0 K.

Radio telescope observations of the 2001 GDS found a globally averaged daytime surface brightness temperature decrease of ~20 K (Gurwell et al., 2005), consistent with the ST cooling in this study (Figure 3a) and TES observations (Smith, 2004). Hanel et al. (1972) used infrared spectroscopy from the Mariner 9 orbiter to examine STs during and after the 1971–1972 GDS; the results support broad dayside cooling and nightside warming, but it is difficult to draw any strong or quantitative conclusions given the limited coverage.

The MSL data set offers a chance for comparison with in situ ST measurements of the 2018 GDS. Guzewich et al. (2019) show, over *L*_S_ = 195–205°, a maximum/minimum ST decrease/increase of 22.8 K/15.1 K, corresponding to a net 3.8‐K decrease. The MGCM at the resolution used cannot explicitly resolve Gale Crater, so an analogue location at the same latitude (−37.5°E, 5.625°S) was chosen. The TI was 294 J·m^−2^·K^−1^·s^−1/2^, compared to the highest published Gale value of 452 J·m^−2^·K^−1^·s^−1/2^, and the average CDOD was 5.3, compared to the MSL‐measured 5.5. The maximum/minimum ST decrease/increase was 23.4 K/20 K, corresponding to a net 1.7‐K decrease. Dayside cooling agrees well, but the MGCM appears to overestimate nightside warming. This is likely due to a lower model TI than that at MSL, which at the time was the high‐TI Vera Rubin Ridge (Edwards et al., 2018), and any local topographic effects not resolved by the MGCM. Dayside STs also start diverging after *L*_S_ ~210° (Figure S4), possibly due to albedo increases from dust deposition causing surface cooling (Fonseca et al., 2018); the MGCM uses a static albedo map. The MGCM's ~250‐km footprint makes meaningful comparison with a point source like MSL difficult; a mesoscale model could offer a better comparison.

Another in situ source is Viking Lander 1 (VL1), which recorded meteorological data from two major storms (Ryan & Henry, 1979); in both cases, maximum/minimum ATs (~1.3‐m altitude) rapidly decreased/increased by ~16 K/~12 K, decreasing on average. Qualitatively, given VL1's relatively high‐TI location, this matches expectations; however, without better knowledge of opacities a more rigorous comparison is not possible.

## 5 Discussion and Conclusions

The MY 34/2018 GDS decreased dayside and increased nightside STs, reducing their diurnal variability. Surprisingly, the diurnally averaged result was a robust and significant net warming over much of the planet. This warming correlated extremely closely with low‐TI regions, which in clear conditions experience rapid nightside cooling; these regions warmed even as diurnally averaged total surface flux decreased, due to significant nightside warming from longwave backscattering, which caused nightside ST increases sufficient to outweigh the dayside cooling. Over regions of higher TI, diurnally averaged STs decreased or remained roughly constant.

Near‐surface air temperatures also showed substantial alteration, driven by the surface temperature changes and the reduced surface‐air temperature gradient. Even in the clear case, heat transport in Mars's atmosphere is dominated by radiation (Barnes et al., 2017; Wolff et al., 2017). Increased dust loading strongly coupled ATs to STs by dramatically increasing radiative absorption (both shortwave and longwave, including of surface emission) in the bottom layers of the atmosphere while reducing shortwave radiative flux at the surface. This resulted in increased ATs at night and even on the dayside for regions where GDS case STs surpassed clear ATs, that is, where the clear‐case surface‐air temperature contrast is greatest.

Interestingly, the MY 34 reanalysis shows less surface warming than the free‐running simulation with the same globally averaged CDOD (Figure 3a); however, MY 34 surface cooling matches the free runs very well. This can be explained in terms of GDS geographical structure. The 2018 GDS was not spatially homogenous; the highest CDOD were over high‐TI regions (Figure 2a), where the nightside warming effect is least. The MY 25 reanalysis, on the other hand, agrees well with the free runs on nightside warming but has stronger dayside cooling. Again, the explanation is geographical: The MY 25 GDS, as represented in the reanalysis (Figure 2d), had a greater latitudinal extent than the MY 34 GDS, with *τ*_vis_ > 1 between 77°S and 66°N versus 69°S and 47°N. This extra area was predominantly high TI and therefore contributed a net cooling effect. Note that TES had limited latitudinal coverage, and so the MY 25 reanalysis used is constructed from spatially kriged observations (Montabone et al., 2015); different GDS decay rates could also potentially affect comparisons. The general conclusion holds, however, that GDS spatial structure is important for its overall radiative effects: Specifically, the magnitude of dust loading over low‐ versus high‐TI areas determines the net ST and AT impacts. The MY 34 GDS also shows noticeable diurnal variation in CDOD (Figure 3), which comes from the variation in MCS CDOD (Kleinböhl et al, this issue); this results in slightly higher dayside cooling/lower dayside warming than in the diurnally uniform CDOD case. The extent to which this is intrinsic variability and not an artifact of MCS dust profile truncation is unclear (Montabone et al., this issue).

One general caveat is that the MGCM uses a static TI map; surface TI has been shown to vary seasonally by up to 200 J·m^−2^·K^−1^·s^−1/2^ and to show day‐night variability (Putzig & Mellon, 2007). GDS have also been shown to cause lasting alteration of albedo and surface TI via dust redistribution (Fenton et al., 2007; Szwast et al., 2006). That said, seasonal TI variations are very small over low‐TI regions, suggesting that net warming over these areas is indeed a robust phenomenon. Nightside STs in the MY 34 reanalysis also agree very well with MCS surface temperature retrievals, suggesting good representation of TI in the MGCM. As noted above though, surface albedo changes may affect representation of dayside STs.

Finally, the nightside warming was more persistent in time than the dayside cooling, which mostly affected peak dayside temperatures. The result was that the warming had an outsized impact on diurnally averaged temperature changes, with more warming in a true diurnal average than in net changes calculated from just two local times. Simulations with varying opacities suggest that a global surface cooling for a GDS with the same structure as the MY 34 event would require a storm opacity of greater than 11; the actual threshold, however, would depend significantly on the storm's geographical structure.

## Supporting information



Supporting Information S1
