# Supplementary material for: Surface Warming During the 2018/Mars Year 34 Global Dust Storm
Source: Geophys Res Lett. 2020 May 6;47(9):e2019GL083936. doi: 10.1029/2019GL083936 (PMC7375149; doi:10.1029/2019GL083936)
Supplement: Supplementary file 1 — Supporting Information S1 [file GRL-47-e2019GL083936-s001.docx]

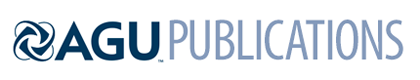


*Geophysical Research Letters*

Supporting Information for

**Surface warming during the 2018/Mars Year 34 Global Dust Storm**

Paul M. Streteer^1^, Stephen R. Lewis^1^, Manish R. Patel^1,2^, James A. Holmes^1^, and David M. Kass^3^

^1^School of Physical Sciences, The Open University, Walton Hall, Milton Keynes MK7 6AA, UK.

^2^Space Science and Technology Department, Science and Technology Facilities Council, Rutherford Appleton Laboratory, Harwell Campus, Didcot, Oxfordshire OX11 0QX, UK.

^3^Jet Propulsion Laboratory, California Institute of Technology, Pasadena, California, USA.

Corresponding author: Paul Streeter ([paul.streeter@open.ac.uk](mailto:paul.streeter@open.ac.uk))

**Contents of this file**

Text S1 to S5

Figures S1 to S7

**Introduction**

This Supporting Information contains additional text to provide more detailed explanation of the Mars Global Climate Model (MGCM) used, the assimilation scheme, how Mars Climate Sounder (MCS) data was processed for assimilation, the radiative transfer scheme, and the justification for the mask applied to the seasonal CO_2_ cap on MCS surface temperature retrieval plots. It also contains plots which compare the MCS surface temperature retrievals to the assimilated MGCM results discussed in the main manuscript, a plot comparing results surface temperature and column dust optical depth (CDOD) results from Mars Science Laboratory (MSL) to the assimilated MGCM results, and plots showing the impact of varying single-scattering albedo (SSA) values for dust on the assimilated MGCM results.

Text S1. Dust transport and scaling in the MGCM

The MGCM used in this study has the capacity to transport dust in three dimensions, and to scale it column-wise according to a prescribed “map”. The source of this map can be dust climatologies (L. Montabone et al., 2015), prescribed dust scenarios, or assimilated column dust observations (Lewis et al., 2007).

The MGCM injects dust at the bottom of the atmosphere at a constant diffusion rate at all locations which are not covered by Mars’ seasonal CO2 cap. Dust is allowed by the MGCM to be vertically mixed by the planetary boundary layer, advected in three dimensions by the semi-Lagrangian advection scheme, and to sediment under the influence of gravity. Dust radiative effects are then calculated. Once this has all taken place, the total column of dust is scaled to match the map; in this case, assimilated column dust optical depth (CDOD) observations from Mars Climate Sounder (MCS).

Dust is transported in the MGCM in the form of a two-moment scheme, using particle mass mixing ratio (“q”) and number density (“N”), with a log-normal size distribution for particle size; from these values and a prescribed value for the density of the dust itself, a particle effective radius can also be calculated (Madeleine et al., 2011).

The dust field used in this work is agnostic regarding the dust vertical distribution; dust can be freely transported in the vertical and only columns are prescribed.

Further details can be found in Madeleine et al. (2011), and in the technical document <http://www.lmd.jussieu.fr/~lmdz/planets/mars/user_manual.pdf>

Text S2. Data assimilation in the MGCM

The MGCM uses a modified version of the UK Met Office’s operational Analysis Correction (AC) scheme (Lorenc et al., 1991), adapted and empirically tuned for the martian atmosphere (Lewis et al., 1997; Lewis et al., 2007). This scheme has the particular advantage of being computationally inexpensive, and can therefore be called every time the model physics is called. Observations are given empirically determined radii of influence in both space and time, with the weight of an observation having a maximum at its valid time and location. The relative weighting given to the observations and the model field variables are also empirically tuned. The aim, as with all data assimilation schemes, is to minimise the cost function: the function describing the degree of “disagreement” between model and observation. In this scheme, this is done by successively correcting the MGCM fields in such a way as to minimise the cost function.

For MCS temperature profiles, the vertical assimilation procedure is performed by first interpolating and re-gridding the profiles to a standard pressure profile matching the intrinsic resolution of the dataset, in this case ~5 km. The model layer thickness are also adjusted to match the re-gridded profile thicknesses, in order to perform a reasonable analysis and to prevent the assimilation from destroying small-scale features near the model bottom, where the spacing between layers is much smaller. Once the analysis has been performed and the model temperature field incremented in the appropriate way, model wind fields are also adjusted (via the thermal wind equation) to match the updated temperature information. This helps maintain a self-consistent circulation and atmospheric state.

For MCS dust columns, spatial increments to the CDOD field are calculated on the MGCM grid. Observations are given weight functions in both distance from actual observation location and time from actual observation time (both in past and future). In addition, observational increments are spread by a correlation scale function: this function has a higher value as the observation weight function is low, and vice versa, meaning that observations which are further spatially and/or temporally from their valid point have a greater footprint. Likewise, observations near their valid time and/or location have smaller spatial footprints. The assimilated CDOD field is then used to prescribe the column opacities of the interactive dust field.

Further details on this implementation can be found in Lewis et al. (2007), Steele et al. (2014), and Holmes et al. (2018; 2019a)

Text S3. Dust quality control and filtering for assimilation into the MGCM

Before assimilation into the MGCM, MCS CDOD values underwent processing and filtering. MCS measures in the infrared (21.6 microns), while the MGCM uses visible values in its dust radiative scheme (600 nm); therefore MCS CDODs are converted via a standard conversion factor of 7.3, the derivation of which is explained in Kleinböhl et al. (2017). A standard filtering is then applied to remove v5.2 MCS dayside CDODs at equatorial latitudes, as these are the most likely to have high profile cut-offs and to be affected by the presence of water ice aerosols, both of which can result in spuriously high CDODs (L. Montabone et al., 2015). This filtering is removed for the period of the 2018 GDS, as a specially-processed v5.3.2 dataset is available for this period which adds extra detectors to the retrieval process to obtain better profiles, and thus more reliable CDOD; dayside clouds are also less likely to be an issue during this period (Montabone et al., this issue).

Text S4. The MGCM radiative transfer scheme and high CDODs

The MGCM uses a two-stream approximation radiative transfer scheme based on the method developed by Toon et al. (1989). The algorithm developed combines various two-stream approximation methods to optimise the stability and accuracy of the calculations. Toon et al. (1989) tested errors in emissivity, transmissivity, and reflectivity in the scheme for optical depths ranging from 0.1 to 100 for four single-scattering albedo (SSA) values. As the dust SSA used in this work is 0.94, we examine errors resulting from use of the highest SSA used by Toon et al. (1989), of 0.9881 (the next highest value was 0.52447) ie. a highly scattering aerosol. For each of emissivity, transmissivity, and reflectivity there was at least one method which remained at or below ~10% error even for optical depths from 10 to 100.

This suggests that even at the very highest CDODs tested in this study, of 15, the results from the radiative transfer scheme should be valid to within a reasonable error, especially given that errors can be close to as high for very “normal” optical depths e.g. of ~1. Therefore any large systematic errors in results at high CDODs are more likely to be due to uncertainties in the dust optical properties, such as SSA (Madeleine et al., 2011), or the CDOD values themselves which have linearly increasing errors (Montabone et al., this issue) than the radiative transfer scheme introducing large errors at high CDODs. Potential errors in SSA are outlined below.

The CDOD range chosen for this work (up to 15) was arbitrarily chosen as covering even the most extreme CDOD values observed by MCS during the 2018 GDS.

Text S5. Rationale for not examining the surface temperatures of the seasonal CO_2_ caps

The physical temperature of the seasonal CO_2_ caps is buffered at the CO2 condensation temperature (which does depend on the surface pressure and thus the elevation and season). This leads to daytime and nighttime temperatures that are identical (McCleese et al., 2008). Likewise, regions covered with seasonal CO_2_ frost are at the same temperature regardless of the Mars year. Furthermore, the extent of the seasonal cap is very consistent from Mars year to Mars year (Calvin et al., 2017; Piqueux et al., 2015). Even a modest amount of dust or other contamination will not matter—the dust grains will very rapidly warm/cool to the local CO2 condensation temperature and have no effect on the surface temperature. (They can cause additional sublimation or deposition, depending on the conditions—this is examined in de la Torre et al. [this issue] for the 2018 GDS.) Thus, comparing the surface temperatures of the seasonal caps between Mars years is very uninteresting since they are essentially the same.

During the *L*_S_=200°-220° period, the seasonal polar caps evolve rapidly, growing/receding by ~10° of latitude (Piqueux et al., 2015). The edges of the caps also show significant latitudinal variability with longitude throughout the season as well (e.g. Figure 7 in Piqueux et al. (2015)). The net result is that whether or not a given location is covered by the seasonal CO_2_ cap (and thus has a buffered temperature) depends on when it is sampled within the Ls window. For the MCS observation, there are differences in the exact timing or location of the surface temperature observations within the bins between the Mars years that are not removed by averaging. Thus, the difference between MY30 and MY34 shows large apparent changes in surface temperatures in the regions of the seasonal polar caps due to the sampling differences.

When the atmosphere above a surface CO_2_ deposit is dusty and warm, uncertainties can be introduced into the retrieval of the surface temperature. Much of the radiance measured at the top of the atmosphere is from the warm airborne dust and little is from the cold CO_2_ surface frost. Uncertainties in the amount of dust or its temperature, especially in regions where the MCS dust profile is extrapolated assuming it is homogenously mixed, can result in uncertainties in the radiance contribution from the dust. This can then result in relatively large errors in the surface temperature. During MY 34, there was significant dust above the southern seasonal polar cap (Kleinbӧhl, et al., this issue). Underestimating the amount of dust will result in surface temperatures noticeably above the CO_2_ condensation temperature.

For these reasons, we have masked off the MCS temperatures and temperature differences (Figs. S1, S2 and S3) with the most equatorward extent of the seasonal CO_2_ polar caps in both hemispheres. The seasonal cap definition we used was from Piqueux et al. (2015). This allows the figures and comparisons to focus on the regions where the surface temperatures are directly affected by the effects of the GDS and are responding to the local dust column.


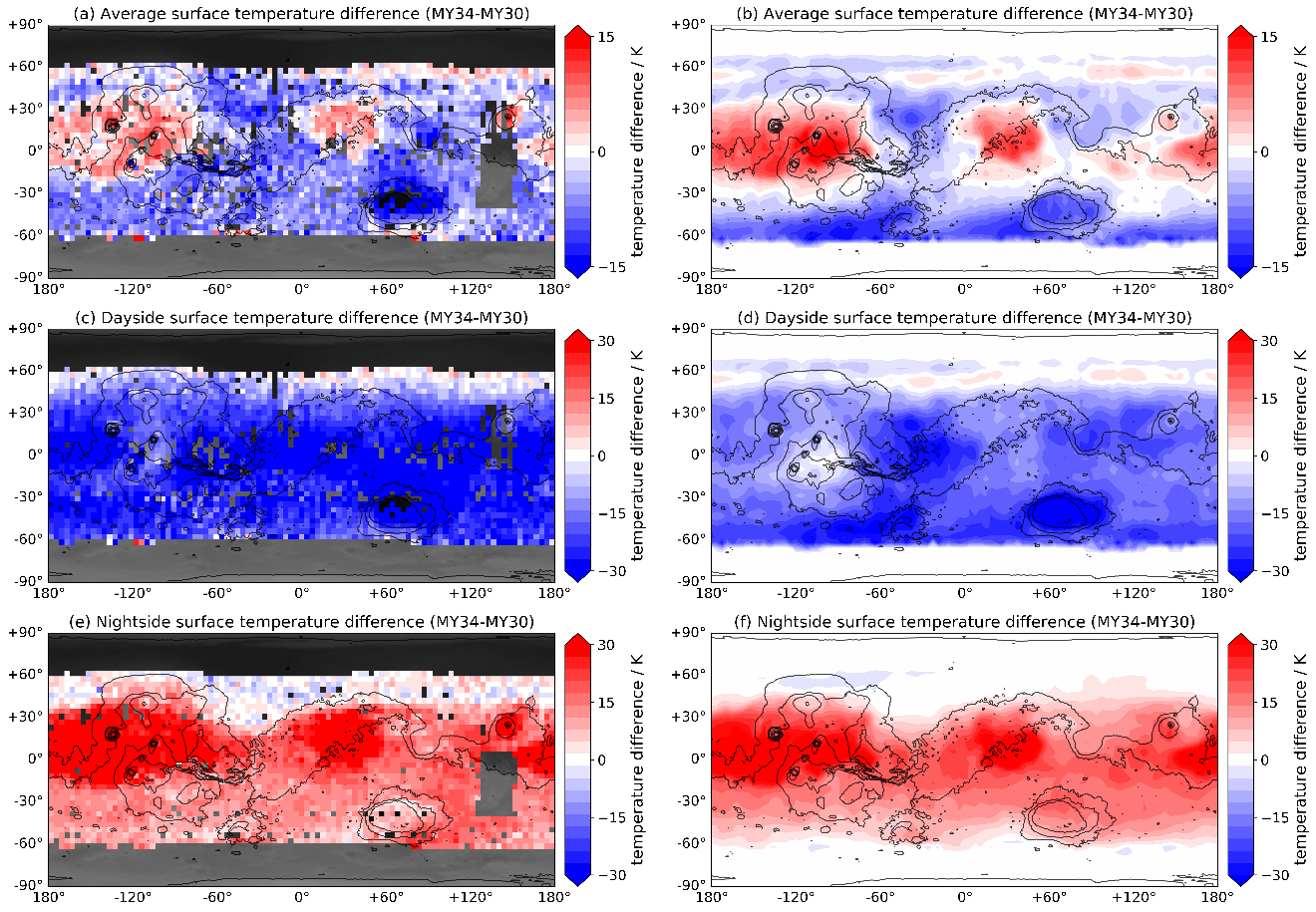


Figure S1. Difference in surface temperatures averaged over *L*_S_=200°-220° between MY 34 and 30 in (left) MCS surface temperature retrievals and (right) the MY 34 reanalysis for (top) the diurnal average (as calculated from dayside and nightside only), (middle) the dayside (3pm), and (bottom) the nightside (3am).


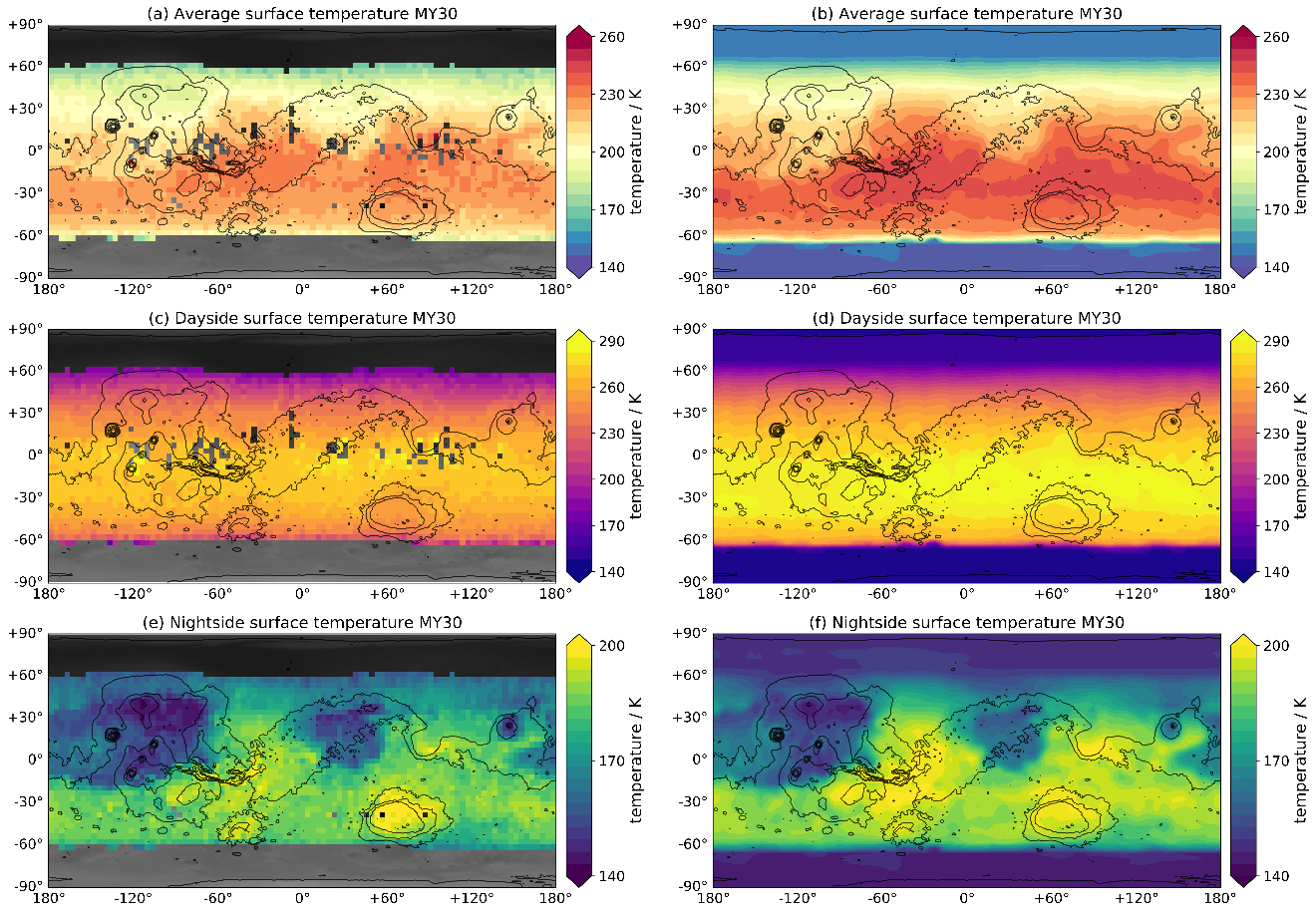


Figure S2. Surface temperatures averaged over *L*_S_=200°-220° in MY 30 for (left) MCS surface temperature retrievals and (right) the MY 34 reanalysis for (top) the diurnal average (as calculated from dayside and nightside only), (middle) the dayside (3pm), and (bottom) the nightside (3am).


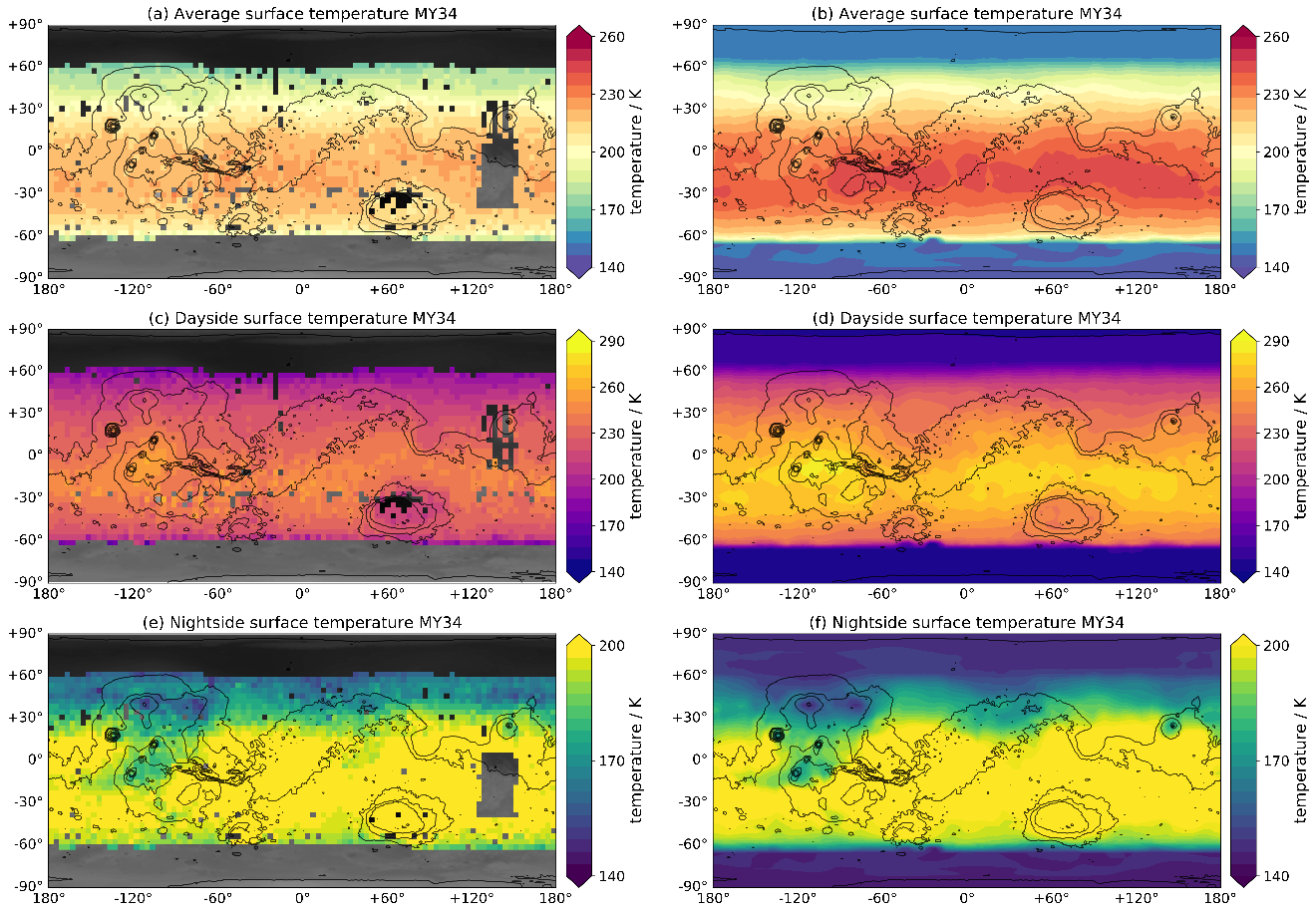


Figure S3. Surface temperatures averaged over *L*_S_=200°-220° in MY 34 for (left) MCS surface temperature retrievals and (right) the MY 34 reanalysis for (top) the diurnal average (as calculated from dayside and nightside only), (middle) the dayside (3pm), and (bottom) the nightside (3am).


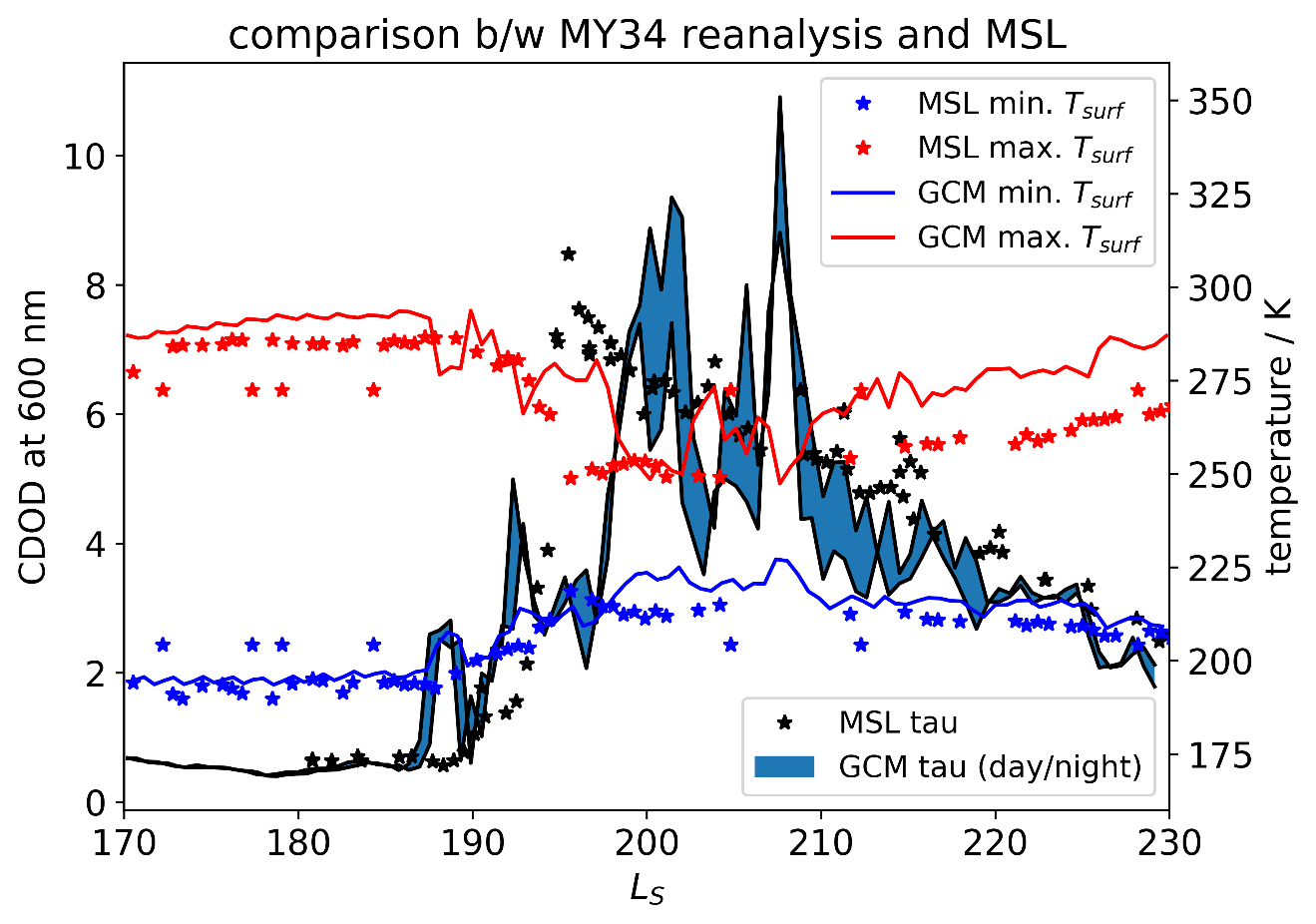


Figure S4. Surface temperatures and CDOD in MY 34 for MSL surface temperature measurements and the MY 34 reanalysis. See manuscript for details of location of reanalysis values.


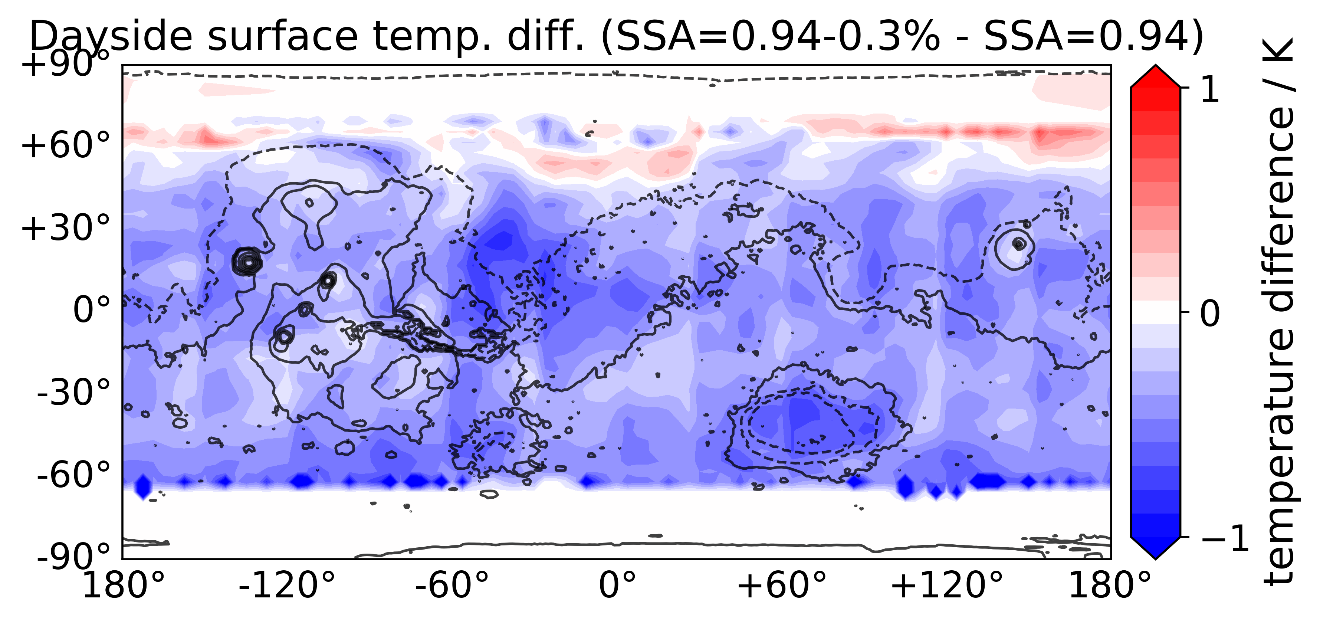


Figure S5. Difference between dayside surface temperatures averaged over *L*_S_=200°-220° in the MY 34 reanalysis between a run with SSA = 0.94 - 0.3% and a run with SSA = 0.94.


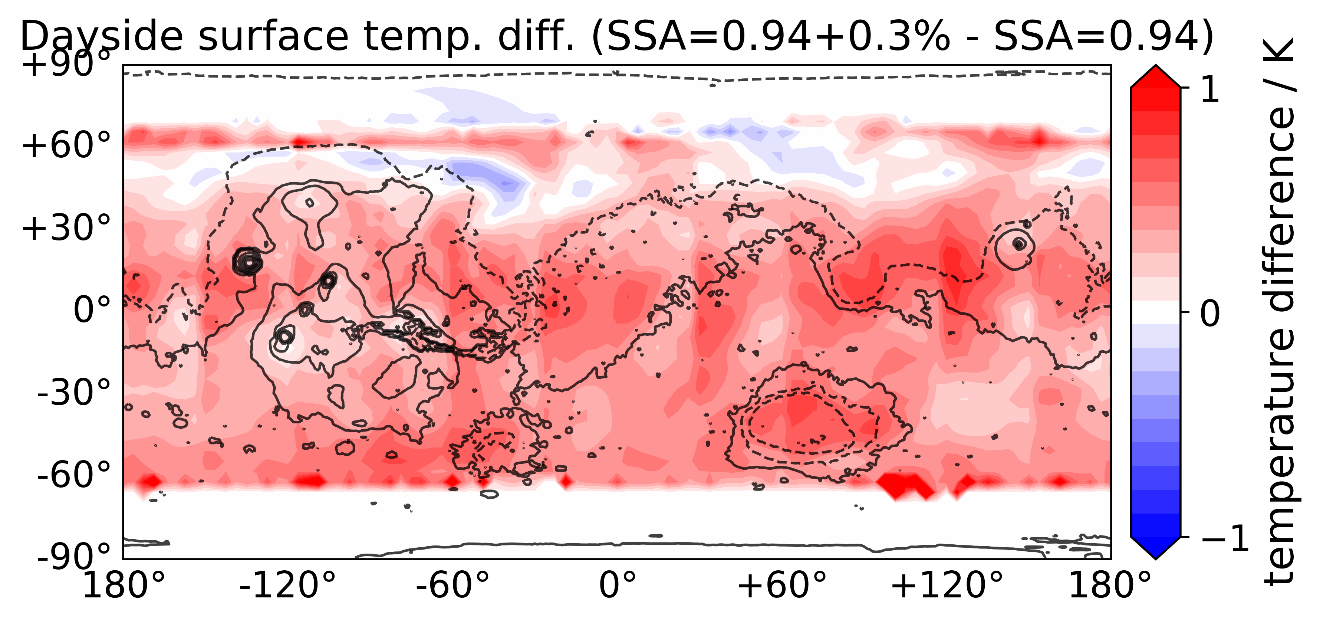


Figure S6. Difference between dayside surface temperatures averaged over *L*_S_=200°-220° in the MY 34 reanalysis between a run with SSA = 0.94 + 0.3% and a run with SSA = 0.94.


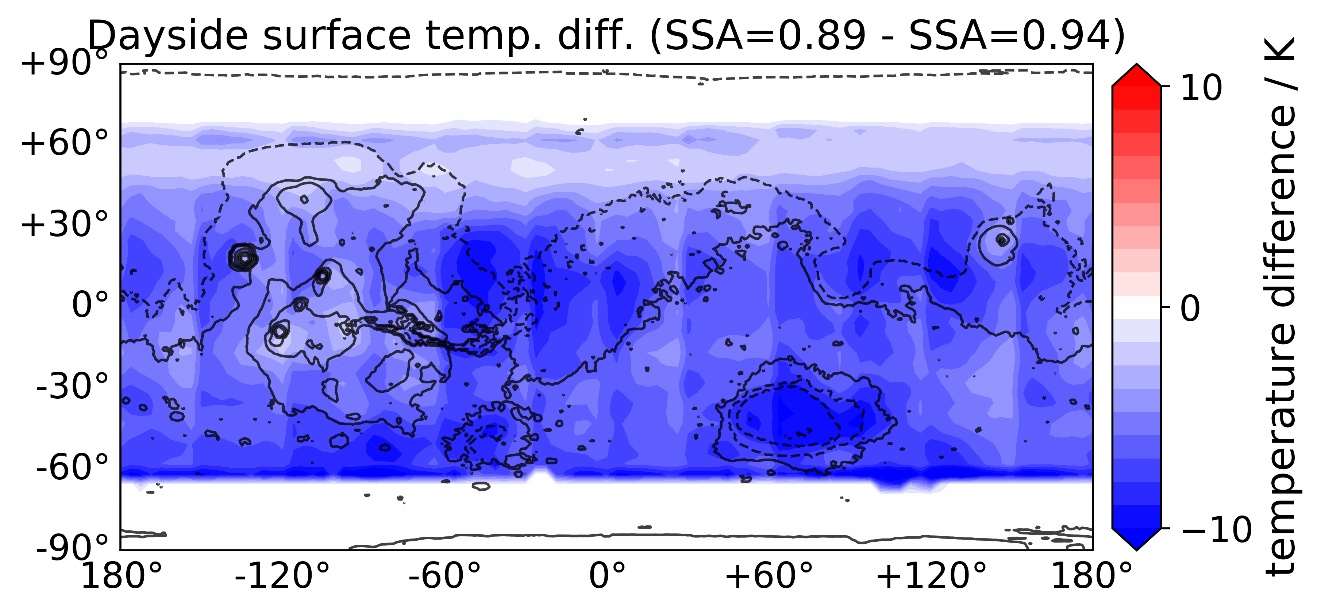


Figure S7. Difference between dayside surface temperatures averaged over *L*_S_=200°-220° in the MY 34 reanalysis between a run with SSA = 0.94 and a run with SSA = 0.94 – 5%. In their work deriving the SSA used in the MGCM, with a value of 0.94, Wolff et al. (2009) discuss earlier, “darker” derived SSA values of approximately 0.89, or ~5% smaller than their derived value. This plot shows the difference for the period of the MY 34 GDS between surface temperatures as calculated by the MGCM using a SSA of 0.89, and of 0.94. The result is colder surface temperatures: for the GDS opacities, as much as 10+ K colder.
